# *N*-3 polyunsaturated fatty acids attenuates triglyceride and inflammatory factors level in h*fat-1* transgenic pigs

**DOI:** 10.1186/s12944-016-0259-7

**Published:** 2016-05-10

**Authors:** Xingxing Liu, Daxin Pang, Ting Yuan, Zhuang Li, Zhanjun Li, Mingjun Zhang, Wenzhi Ren, Hongsheng Ouyang, Xiaochun Tang

**Affiliations:** Jilin Provincial Model Animal Engineering Research Center, College of Animal Sciences, Jilin University, Xi’an Road, 5333#, Jilin, 130062 China

**Keywords:** *n*-3 polyunsaturated fatty acids, Transgenic pig- fat-1, Inflammation, Triglyceride

## Abstract

**Background:**

The consumption of *n*-3 polyunsaturated fatty acids (PUFAs) is important to human health, especially in cases of cardiovascular disease. Although beneficial effects of *n*-3 PUFAs have been observed in a number of studies, the mechanisms involved in these effects have yet to be discovered.

**Methods:**

We generated h*fat-1* transgenic pigs with traditional somatic cell nuclear transfer (SCNT) technology. The fatty acid composition in ear tissue of pigs were detected with gas chromatography. The cholesterol, triglycerides (TAG) and inflammation mediators in circulation were investigated.

**Results:**

The h*fat-1* transgenic pigs were developed which accumulate high levels of *n*-3 PUFAs than wild-types pigs. Gas chromatography results demonstrated that the total *n*-3 PUFAs in the ear tissues of the transgenic founders were 2-fold higher than the wild-type pigs. A lipid analysis demonstrated that the levels of TAG in the transgenic pigs were decreased significantly. The basal levels of the inflammation mediators tumor necrosis factor-α (TNF-α), monocyte chemoattractant protein-1 (MCP-1) and interleukin-6 (IL-6) in transgenic pigs were inhibited markedly compared with the wild-type pigs.

**Conclusions:**

These results suggest that *n*-3 PUFAs accumulation in vivo may have beneficial effects on vascular and h*fat-1* transgenic pigs may be a useful tool for investigating the involved mechanisms.

## Background

N-3 polyunsaturated fatty acids (PUFAs) are important dietary fatty acids, including alpha-linolenic acid (ALA), eicosapentaenoic acid (EPA) and docosahexaenoic acid (DHA). ALA is enriched in seed oil and can be obtained directly through diets [1]. EPA and DHA can be converted from ALA through desaturation-chain elongation pathway in mammals [2, 3]. Although young women has greater conversion capacity of ALA to DHA than men, the synthesis efficiency is still limited [4–8]. Thus, the primary source of this fatty acids for humans is dietary supplementation.

However, *fat-1* gene in caenorhabditis elegans encodes an *n*-3 fatty acids desaturase which are able to convert *n*-6 to *n*-3 fatty acids by adding a double bond into n-6 fatty acids at the *n*-3 hydrocarbon position [9, 10]. Researchers developed transgenic mice, pigs and cows carrying the *fat-1* gene previously and observed higher *n*-3 PUFAs accumulation in tissues [11–14].

A number of observational studies, clinical trials, and in vitro studies have demonstrated that the consumption of *n*-3 PUFAs is beneficial in the management of cardiovascular disease, including atherosclerosis, severe arrhythmias, and thrombosis, and decrease the incidence of cardiac arrest and sudden death [15–19]. Moreover, the American Heart Association (AHA) recommends that an intake of approximately 500 mg/day of *n*-3 PUFAs be used to prevent cardiovascular disease (CVD) and that patients with CVD should have an intake of 2–4 g/day [20–22]. In animals, accumulation of *n*-3 PUFAs in *fat*-1 transgenic mice decreased cholesterol level and body weight [23], attenuated vascular inflammation which represented by CD68 and neointimal hyperplasia [24], protected against global ischemia injury [25]. Moreover, decreased ratio of *n*-6/*n*-3 in *apoE*^*-/-*^*/fat-*1 mice reduced aortic lesion area significantly than that of apoE^-/-^ littermates [26].

Although rodents have been widely used in medical research as disease models, some of information that come from rodents still can not be replicated and has failed to translate into clinical practice. The pig has a heart size similar to that of humans, has a similar coronary circulation and can develop spontaneous atherosclerotic lesions [27–29]. Thus, to mimic the role of *n*-3 PUFAs in humans, we developed h*fat-1* transgenic pigs and investigate the changes in cholesterol, TAG, and inflammation caused by the metabolism of *n*-3 PUFAs.

## Methods

### Generation of transgenic pigs

**Fig. 1:**
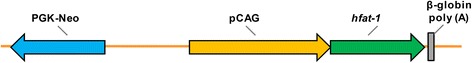
The expression cassette, mainly including the pCAG promoter, humanized *fat-1* gene, and a neomycin selection cassette

The h*fat-1* expression plasmid with CAG promoter was donated by Dr. Dai (Nanjing Medical University, Jiangsu, China). The *fat-1* gene is from C. elegans (GenBank: NM_001028389) and was optimized for mammal expression. The plasmid (Fig. 1) was linearized and transfected into primary fetal fibroblasts that were isolated from a Song-liao black pig using Fugene HD (Roche, Basel, Switzerland). After 24 h of transfection, the cells were split 1:36 and cultured in selection medium containing 500 μg/ml G418 antibiotic (Merck, Shanghai, China). After 10 days of selection, the surviving cell colonies were propagated in 24-well plates with 200 μg/ml G418 antibiotic for another 4 days. G418 resistant clones were analyzed with PCR assays (Tiangen, Beijing, China) using the following primers: h*fat-1* identi forward (GGA CCT GGT GAA GAG CAT CCG) and h*fat-1* identi reverse (GCC GTC GCA GAA GCC AAA C) located in the h*fat-1* gene and neo identi forward (ATG ATT GAA CAA GAT GGA TTG CAC GC) and neo identi reverse (TCA GAA GAA CTC GTC AAG AAA GGC GAT AG) located in the neomycin gene. The PCR assays were conducted using 30 cycles of denaturation at 94 °C for 30 s, annealing at 55 and 50 °C for 30 s, and extension at 72 °C for 90 s. The PCR products were analyzed by electrophoresis and sequencing to confirm the integrity of the h*fat-1* gene within the pig genome in the G418 resistant clones. The five identified positive colonies were selected as donor cells to perform somatic cell nuclear transfer (SCNT) and embryo transfer as previously described [30]. The pigs housed in a 18–20 °C environment in winter with free access to water and were fed three times per day (morning, 6:00; noon, 13:00; evening, 17:30) with a diet containing crude protein 15 % and crude fat 4 %. All the animal experiments and maintenance protocols were in accordance with the Guide for the Care and Use of Laboratory Animals and were approved by Jilin University.

### Genotype and tissue fatty acid analysis of the founders

Genomic DNA from the cloned piglets was extracted from the tails and subjected to PCR analysis to confirm the insertion of the h*fat-1* gene using the h*fat-1* identi forward and reverse primers. The PCR products were analyzed by electrophoresis and sequencing. The fatty acid composition in the ears which selected according to the previous research of the transgenic founders was analyzed using gas chromatography–mass spectrometry (GC–MS) (6890 N, Agilent, USA) method as described in other reports [13, 31, 32]. The initial temperature of the program was 160 °C for 2 min, then increased at a rate of 1 °C/min to 210 °C for 50 min. The fatty acid concentration is presented as a percentage of the total fat in the ears. Each value represents the mean ± standard deviation. Each sample measurement was performed three times.

### Quantitative real-time PCR

Blood was collected from pigs using anticoagulant EDTA tubes, and peripheral blood mononuclear cells (PBMCs) were isolated from the whole blood with Histopaque-1077(Sigma Inc., USA) according to the manufacturer’s instructions. Briefly, add 5 ml Histopaque-1077 into 50 ml tube, carefully add 5 ml blood onto Histopaque-1077, centrifuge at 400 × g for 30 min, carefully transfer the opaque interface which containing PBMCs into a new tube. Total RNA was isolated from the PBMCs (five transgenic pigs and three wild-type pigs) using the TRIzol-A^+^ reagent (Tiangen, Beijing, China) according to the manufacturer’s instructions. First-strand cDNAs were synthesized from 1 μg of total RNA using a BioRT cDNA first stand synthesis kit (Bioer, Hangzhou, China), and the samples were analyzed with a Bioeasy SYBR green I real-time PCR kit (Bioer, Hangzhou, China). The detection of inflammation mediators were performed using the following primers: pMCP-1 forward (TCACCAGCAGCAAGTGTCCT) and pMCP-1 reverse (ATGTGCCCAAGTCTCCGTTT); pIL-6 forward (TGGGTTCAATCAGGAGAC) and pIL-6 reverse (CTGACCAGAGGAGGGAAT); pTNF-α forward (CGCATCGCCGTCTCCTACCA) and pTNF-α reverse (TGCCCAGATTCAGCAAAGTCCA). The gene expression was normalized against the internal control (β-actin).

### Plasma lipid and lipoprotein analysis

After 16 h of food deprivation, 5 ml of blood was obtained from precaval vein of each pig. Centrifuge at 1000 × g for 10 min to obtain plasma. The TAG, total cholesterol (TC), HDL-C and LDL-C levels in each sample were determined by corresponding kit (ERKN, Zhejiang, China) and analyzed using Beckman coulter UniCel DxC 800 Synchron (Beckman coulter, USA) by No. 208 Hospital of the People’s Liberation Army.

### Statistical analysis

Data in Figs. 3 and 4 are expressed as the mean ± SEM. Comparisons were performed using an unpaired two-tailed Student’s *t*-test unless otherwise specified. Data were analyzed using GraphPad Prism6. *P < 0.05* was considered to be statistically significant.

## Results

### Generation of h*fat-1* transgenic pigs

**Fig. 2:**
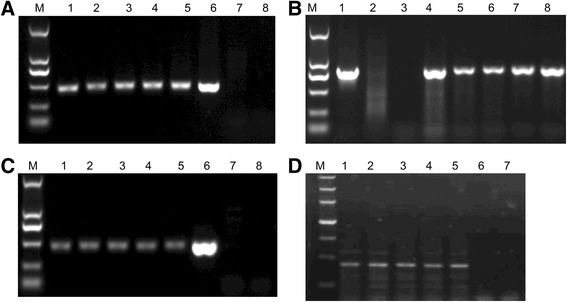
Identification of G418-resistant clones and h*fat-1* transgenic pigs. Analyse of the h*fat-1* gene and neomycin gene integration into the pig genome in the G418-resistant clones were performed using a PCR assay with the primer pairs of h*fat-1* identi (458 bp) (**a**) and neo identi (790 bp) (**b**). **a** 1–5, five G418-resistant clones, on which SCNT was individually performed in the following procedure. 6, h*fat-1* plasmid as a positive control. 7–8, negative controls. **b** 1, h*fat-1* plasmid as a positive control. 2–3, negative controls. 4–8, five G418-resistant clones. The h*fat-1* gene integration into the pig genome and transcription in the transgenic pigs were analyzed using PCR (**c**) and RT-PCR (**d**), respectively, with the h*fat-1* identi primer pair (458 bp). **c** 1–5, five h*fat-1* transgenic pigs. 6, h*fat-1* plasmid as a positive control. 7–8, two wild-type pigs. **d** 1–5, five h*fat-1* transgenic pigs. 6–7, two wild-type pigs

Unlike the development of transgenic mice which use embryonic stem cells (ES) for preselection of gene insertion [33], primary embryonic pig fibroblasts were employed for transfection with a linearized h*fat-1* expression plasmid. The screening for positive cells was performed in G418-containing culture medium for 10 days; 50 G418 -resistant clones were picked and analyzed via PCR for the integration of h*fat-1* cDNA. Thirty clones exhibited both the expected 458 bp band of h*fat-1* and the 790 bp band of the neomycin sequence (data not shown); 5 of these clones were selected for presentation in this paper and for individually performing somatic SCNT in the following step (Fig. 2a and b).

On average, 248 reconstructed embryos from each positive clone were transferred into each naturally cycling gilt, and totally five recipients. At days 25–28, 2 recipients (40 %) became pregnant, as determined by ultrasound scanning. One of these aborted at 29 days, and one pregnancy went to term (20 %). Five piglets were born by natural delivery. The cloning efficiency was 0.4 % (No. of piglets/No. of embryos transferred). The genomic PCR assay was performed using DNA extracted from the tail tissue, and the results show that all five piglets were positive for the h*fat-1* transgene (Fig. 2c).

### Accumulation of *n*-3 PUFAs in the transgenic pigs

To examine whether h*fat-1* was transcribed in the transgenic pigs, reverse transcription PCR was performed using cDNA obtained from the tail. The results show that h*fat-1* was transcribed in all five of h*fat-1* positive transgenic pigs (Fig. 2d). To investigate whether the transfected h*fat-1* gene was functional in the transgenic pigs, gas chromatography was performed to determine the composition of the PUFAs. The fatty acid profiles in Table 1 were derived from the ear tissues of five transgenic pigs and three wild-type pigs. The total *n*-3 PUFAs in the ear tissues of the transgenic founders were 2-fold higher than those of the wild-type pigs. However, the *n*-6 PUFAs of the transgenic founders were not significantly altered, and the ratio of *n*-6/*n*-3 fatty acids was reduced 2-fold in the transgenic founders compared with the wild-type pigs.

**Table 1:** Statistical analyses were performed using an unpaired two-tailed Student’s *t*-test

Fatty acid in ears	Transgenic piglets (*n* = 5)	Wild-type piglets (*n* = 3)
18:3 *n*-3 (%)	2.25 ± 0.15	1.12 ± 0.09
20:5 *n*-3 (%) (EPA)	2.71 ± 0.72	1.09 ± 0.12
22:5 *n*-3 (%)	1.23 ± 0.21	0.51 ± 0.06
22:6 *n*-3 (%) (DHA)	1.15 ± 0.17	0.38 ± 0.08
18:2 *n*-6 (%)	3.28 ± 0.25	5.72 ± 0.64
20:4 *n*-6 (%)	5.78 ± 0.57	5.65 ± 0.39
22:5 *n*-6 (%)	1.19 ± 0.22	2.22 ± 0.16
Total *n*-3 FA (%)^a^	7.34 ± 0.76	3.10 ± 0.06
Total *n*-6 FA (%)	10.25 ± 2.28	13.59 ± 0.24
*n*-6/*n*-3 ratio^a^	1.81 ± 0.41	4.38 ± 0.64

^a^ *P* < 0.01 Table 1 *n*-3 and *n*-6 fatty acids concentration and n-6/n-3 ratios in ear samples from h*fat-1* transgenic pigs and wild-type pigs

### Lipid analysis of the h*fat-1* transgenic pigs

**Fig. 3:**
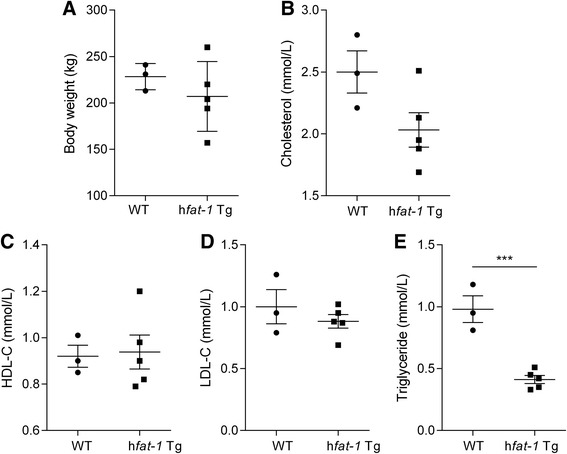
The body weight and plasma characters. **a** The body weight of wild-type and h*fat-1* transgenic pigs at 18 month. Plasma was isolated from the blood of the transgenic and wild-type pigs after 16 h of food deprivation and assayed for TC (**b**), HDL-C (**c**), LDL-C (**d**), and TG (**e**). ***, *p* < 0.001

There were no difference of body weight between wild-type and transgenic pigs (Fig. 3a). To examine whether an abundance of *n*-3 PUFAs is beneficial to the vasculature, the cholesterol and TAG concentrations in the blood of one-year-old transgenic and wild-type pigs were measured. The results demonstrated that TC was not different between transgenic and wild-type pigs in fasting states (Fig. 3b). HDL-C and LDL-C levels were also not significantly altered under the same conditions (Fig. 3c and d). However, the level of TAG in h*fat-1* transgenic pigs was decreased significantly compared with the wild-type pigs (Fig. 3e).

### Inflammatory factors analysis of the h*fat-1* transgenic pigs

**Fig. 4:**
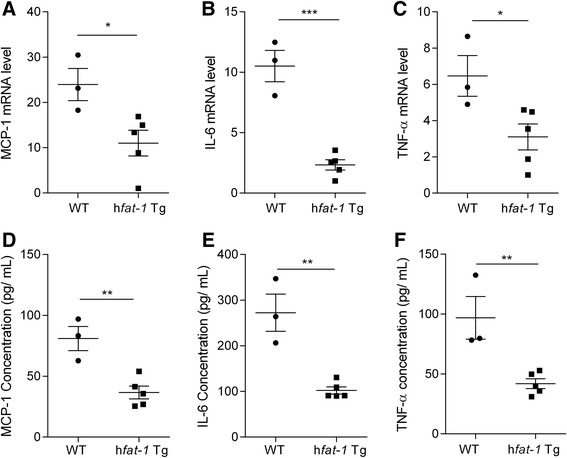
The levels of proinflammatory cytokines in h*fat-1* transgenic pigs. Total RNA was isolated from the blood monoyte of transgenic and wild-type pigs and qRT-PCR was performed. **a-c** MCP-1, IL-6 and TNF-α mRNA level in transgenic and wild-type pigs. Plasma was isolated from the blood of the transgenic and wild-type pigs and MCP-1, IL-6 and TNF-α concentration were analysed by Elisa. **d-f** MCP-1, IL-6 and TNF-α concentration in transgenic and wild-type pigs. All data are presented as the mean ± SEM (five transgenic pigs and three wild-type pigs) *, *P* < 0.01. **, *P* < 0.001

Because cardiovascular disease correlates with chronic inflammation, the transcription and concentration of inflammation genes were measured with a real-time PCR assay using PBMCs cDNA and with Elisa using plasma of the transgenic and wild-type pigs. The result shows that MCP-1, IL-6 and TNF-α mRNA levels were reduced obviously in the transgenic pigs compared with the wild-type pigs (Fig. 4a, b and c). In accordance with the transcription level, the concentrations of MCP-1, IL-6 and TNF-α were decreased significantly as well. These results indicate that an abundance of *n*-3 PUFAs is beneficial for maintaining a lower level of inflammation in the body, which is consistent with previous research [24, 26, 34].

## Discussion

*N*-3 PUFAs are important for human health. Moreover, meat products usually have less *n*-3 and more *n*-6 PUFAs. The imbalance of *n*-6/*n*-3 intake contribute to the development of CVD [18, 35]. In this study, we introduced the *n*-3 fatty acid desaturase encoding gene *fat-1* from *C .elegans* into the pig using SCNT technology, and the gene was successfully expressed in the transgenic pigs. The cholesterol, TAG and inflammatory cytokines of h*fat*-1 transgenic pigs were determined.

The h*fat-*1 transgenic pigs in our study were cloned from one G418 resistant clone for more stable gene expression and accumulation of fatty acids. The concentrations of total *n*-3 PUFAs in the ear tissues of our transgenic founders were two-fold higher than in the wild-type pigs, but the *n*-6 PUFAs were less altered. Additionally, the EPA and DHA levels showed a 2-fold and 3-fold increase in the transgenic founders, respectively. Consequently, the ratio of *n*-6/*n*-3 fatty acids was decreased two-fold in the transgenic founders compared with wild-type pigs. In a previous study, Saeki et al. got 20 % more linoleic acid (omega-6 PUFA) in adipose tissue in △12 fatty acid desaturase gene transgenic pigs [36]. Lai et al. reported that the levels of *n*-3 PUFAs in the tail tissues of transgenic pigs were three-fold higher than those in wild-type pigs, and the ratio of *n*-6/*n*-3 fatty acids was reduced five-fold in the transgenic pigs [14]; Zhou et al. reported that the level of *n*-3 PUFAs in the muscle tissues of *cbr-fat*-1 transgenic pigs was six-fold higher than that of the wild-type pigs and the ratio of *n*-6/*n*-3 fatty acids was reduced 10.5 fold in the transgenic founders [11]. However, the accumulation of *n*-3 PUFAs is not as high and the ratio of *n*-6/*n*-3 PUFAs is not altered as much in our results as in previous reports.

In *fat-1* transgenic mice, decreased HDL-C, LDL-C, TC and TAG were observed, but the body weight is not uniform [23, 37]. In the h*fat-*1 transgenic pig, there was no difference observed in body weight compared to wild-type pigs. A high level of TAG is supposed to be an independent risk factor of coronary heart disease (CHD) according to AHA reports [20, 38]. As pigs, the basal TAG levels in Song-liao black is higher compared with other miniature pigs (data not shown). In the h*fat-1* transgenic pigs, the accumulation of *n*-3 PUFAs lowered the TAG levels significantly in circulation in a fasting states compared with the levels in the wild-type pigs. In addition, plasma TC and HDL-C were not changed in the transgenic pigs. Plasma LDL-C was not statistically decreased. Therefore, the lowered TAG levels might exert some protective effects in the h*fat-1* transgenic pig.

Chronic inflammation plays an important role in the progression of cardiovascular disease. A number of studies have reported that EPA and DHA or fish oil inhibit endotoxin-induced IL-6, TNF-α and IL-1∙production in vitro and in vivo [39–42]. In the h*fat-1* transgenic pigs, the basal mRNA levels of the inflammation mediators IL-6, TNF-α and MCP-1 were inhibited compared with those of the wild-type pigs. In accordance with the mRNA level, the concentrations of MCP-1, IL-6 and TNF-α were decreased significantly as well. Therefore, the anti-inflammatory effects of *n*-3 PUFAs may contribute to the lower inflammation levels in mammals.

Although the accumulation of *n*-3 PUFAs in our transgenic pigs was not as high as previous reports, the TAG and inflammatory factor levels were decreased indeed under similar total *n*-3 plus *n*-6 PUFAs levels. The results illustrated that moderate alteration of *n*-6/*n*-3 PUFAs ratio is enough to affect metabolism in mammals.

## Conclusions

We obtained h*fat-1* transgenic pigs that accumulate higher levels of *n*-3 PUFAs than do wild-type pigs. The *n*-3 PUFAs in the ear tissues of the transgenic founders were 2-fold higher than those of the wild-type pigs. TAG levels in the h*fat-1* transgenic pigs were decreased significantly, whereas TC, HDL-C and LDL-C were not statistically different. However, the levels of the inflammatory mediators IL-6, TNF-α and MCP-1 were decreased significantly as well. Therefore, *n*-3 PUFAs supplementation in human daily life may have protective effects on vascular.

## Abbreviations

IL-6: interleukin-6
MCP-1: monocyte chemoattractant protein-1
PBMCs: peripheral blood monouclear cells
PUFAs: polyunsaturated fatty acids
TAG: triglycerides
TNF-α: tumor necrosis factor-α
